# 1-Isobutyl-4-meth­oxy-1*H*-imidazo[4,5-*c*]quinoline

**DOI:** 10.1107/S1600536811031801

**Published:** 2011-08-11

**Authors:** Hoong-Kun Fun, Wan-Sin Loh, Dinesha, Reshma Kayarmar, G. K. Nagaraja

**Affiliations:** a X-ray Crystallography Unit, School of Physics, Universiti Sains Malaysia, 11800 USM, Penang, Malaysia; b Department of Chemistry, Mangalore University, Karnataka, India; c Sequent Scientific Limited, Baikampady, New Mangalore, India

## Abstract

In the title compound, C_15_H_17_N_3_O, the 1*H*-imidazo[4,5-*c*]quinoline ring system is approximately planar, with a maximum deviation of 0.036 (1) Å. The C—N—C—C torsion angles formed between this ring system and the isobutyl unit are −99.77 (16) and 79.71 (17)°. In the crystal, inter­molecular C—H⋯O hydrogen bonds link the mol­ecules into chains along the *c* axis.

## Related literature

For background to quinolines and their microbial activity, see: Crozat & Beutler (2004 ▶); Stringfellow & Glasgow (1972 ▶); Miller *et al.* (1999 ▶); Hemmi *et al.* (2002 ▶). For related structures, see: Loh *et al.* (2011**a* ▶,b* ▶).
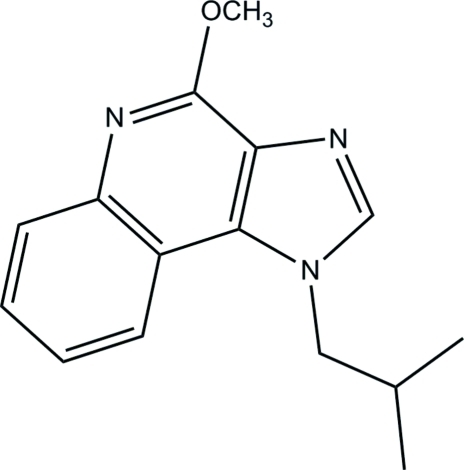


## Experimental

### 

#### Crystal data

C_15_H_17_N_3_O
*M* *_r_* = 255.32
Monoclinic, 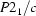

*a* = 7.4196 (8) Å
*b* = 18.910 (2) Å
*c* = 10.4112 (14) Å
β = 110.568 (2)°
*V* = 1367.6 (3) Å^3^
*Z* = 4
Mo *K*α radiation
μ = 0.08 mm^−1^
*T* = 297 K
0.40 × 0.31 × 0.13 mm

#### Data collection

Bruker SMART APEXII DUO CCD area-detector diffractometer
Absorption correction: multi-scan (*SADABS*; Bruker, 2009 ▶) *T*_min_ = 0.969, *T*_max_ = 0.989
12741 measured reflections
3463 independent reflections
2386 reflections with *I* > 2σ(*I*)
*R*_int_ = 0.023

#### Refinement

*R*[*F* ^2^ > 2σ(*F* ^2^)] = 0.042
*wR*(*F* ^2^) = 0.127
*S* = 1.04
3463 reflections
175 parameters
H-atom parameters constrained
Δρ_max_ = 0.12 e Å^−3^
Δρ_min_ = −0.18 e Å^−3^

### 

Data collection: *APEX2* (Bruker, 2009 ▶); cell refinement: *SAINT* (Bruker, 2009 ▶); data reduction: *SAINT*; program(s) used to solve structure: *SHELXTL* (Sheldrick, 2008 ▶); program(s) used to refine structure: *SHELXTL*; molecular graphics: *SHELXTL*; software used to prepare material for publication: *SHELXTL* and *PLATON* (Spek, 2009 ▶).

## Supplementary Material

Crystal structure: contains datablock(s) global, I. DOI: 10.1107/S1600536811031801/wn2444sup1.cif

Structure factors: contains datablock(s) I. DOI: 10.1107/S1600536811031801/wn2444Isup2.hkl

Supplementary material file. DOI: 10.1107/S1600536811031801/wn2444Isup3.cml

Additional supplementary materials:  crystallographic information; 3D view; checkCIF report

## Figures and Tables

**Table 1:** Hydrogen-bond geometry (Å, °)

*D*—H⋯*A*	*D*—H	H⋯*A*	*D*⋯*A*	*D*—H⋯*A*
C10—H10*A*⋯O1^i^	0.93	2.39	3.3052 (16)	169

Symmetry code: (i) 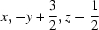.

**Fig. 1.**
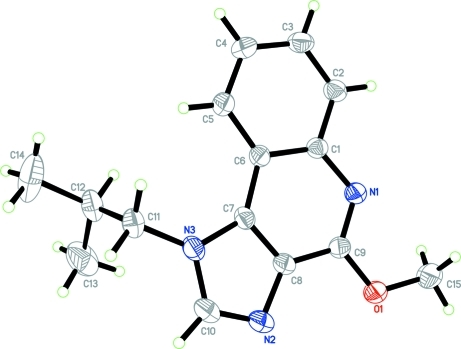
The molecular structure of the title compound, showing 30% probability displacement ellipsoids and the atom-numbering scheme. Hydrogen atoms are shown as spheres of arbitrary radius.

**Fig. 2.**
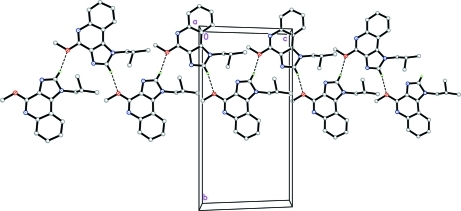
The crystal packing of the title compound, viewed along the a axis, showing the chains along the c axis. Hydrogen bonds are indicated by dashed lines.

## supplementary crystallographic information

### Comment

The quinoline scaffold is prevalent in a variety of pharmacologically active synthetic and natural products. Long before endosomal TLR7 was discovered to serve as the primary sensor for short, single-stranded, GU-rich RNA sequences (ssRNA), mainly of viral origin (Crozat & Beutler, 2004), a number of small molecules were synthesized and evaluated in the 1970's and 1980's for antiviral activities owing to their pronounced type I interferon (IFN-*R* and -β) inducing properties (Stringfellow & Glasgow, 1972). Although the mechanisms of innate immune stimulation of several of these compounds (such as tilorone14 and bromopirone16) remain yet to be formally elucidated, the members of the 1*H*-imidazo[4,5-*c*]quinolines were found to be good type I IFN inducers in human cell-derived assays and FDA approval was obtained in 1997 for imiquimod for the treatment of basal cell carcinoma and actinic keratosis (Miller *et al.*, 1999). It was not until 2002, however, that the mechanistic basis of IFN induction by the imidazoquinolines was found to be a consequence of TLR7 engagement and activation (Hemmi *et al.*, 2002). We have earlier reported the crystal structures of 1-isobutyl-*N*,*N*-dimethyl-1*H*-imidazo[4,5-*c*]quinolin- 4-amine and 4-hydrazinyl-1-isobutyl-1*H*-imidazo[4,5-*c*]quinoline (Loh *et al.*, 2011**a*,b*). Following on from these, we have synthesized 1-isobutyl-4-methoxy-1*H*-imidazo[4,5-*c*]quinoline.

In the title compound (Fig. 1), the 1*H*-imidazo[4,5-*c*]quinoline ring system (C1–C7/N3/C10/N2/C8/C9/N1) is approximately planar with a maximum deviation of 0.036 (1) Å at atom C8. The torsion angle, C10—N3—C11—C12, formed between this ring system and the isobutyl unit is -99.77 (16)°; the torsion angle C7—N3—C11—C12 is 79.71 (17)°. Bond lengths and angles are within the normal ranges and are comparable to those in the related crystal structures (Loh *et al.*, 2011**a*,b*).

In the crystal packing (Fig. 2), the intermolecular C10—H10A···O1 hydrogen bonds (Table 1) link the molecules into chains along the *c* axis.

### Experimental

To a solution of 4-chloro-1-(2-methylpropyl)-1*H*-imidazo[4,5-*c*] quinoline (0.1 mol) in methanol (30 ml) was added a solution of sodium methoxide (0.01 mol) in methanol (10 ml) and the mixture was stirred for 1 h. The reaction mixture was heated under reflux for 12 h, concentrated and poured into crushed ice. The resultant solid was filtered, dried and recrystallized using a mixture of DMF and water (1:1). *M. p.* = 493–495 K.

### Refinement

All H atoms were positioned geometrically and refined using a riding model; C—H = 0.93 to 0.98 Å; *U*_iso_(H) = x*U*_eq_(C), where x = 1.5 for methyl H and 1.2 for all other H atoms. A rotating group model was applied to the methyl groups.

### Crystal data

**Table:**

C_15_H_17_N_3_O	*F*(000) = 544
*M**_r_* = 255.32	*D*_x_ = 1.240 Mg m^−^^3^
Monoclinic, *P*2_1_/*c*	Mo *K*α radiation, λ = 0.71073 Å
Hall symbol: -P 2ybc	Cell parameters from 3364 reflections
*a* = 7.4196 (8) Å	θ = 2.9–28.4°
*b* = 18.910 (2) Å	µ = 0.08 mm^−^^1^
*c* = 10.4112 (14) Å	*T* = 297 K
β = 110.568 (2)°	Plate, colourless
*V* = 1367.6 (3) Å^3^	0.40 × 0.31 × 0.13 mm
*Z* = 4	

### Data collection

**Table:**

Bruker SMART APEXII DUO CCD area-detector diffractometer	3463 independent reflections
Radiation source: fine-focus sealed tube	2386 reflections with *I* > 2σ(*I*)
graphite	*R*_int_ = 0.023
φ and ω scans	θ_max_ = 28.6°, θ_min_ = 2.2°
Absorption correction: multi-scan (*SADABS*; Bruker, 2009)	*h* = −7→9
*T*_min_ = 0.969, *T*_max_ = 0.989	*k* = −25→25
12741 measured reflections	*l* = −13→10

### Refinement

**Table:**

Refinement on *F*^2^	Primary atom site location: structure-invariant direct methods
Least-squares matrix: full	Secondary atom site location: difference Fourier map
*R*[*F*^2^ > 2σ(*F*^2^)] = 0.042	Hydrogen site location: inferred from neighbouring sites
*wR*(*F*^2^) = 0.127	H-atom parameters constrained
*S* = 1.04	*w* = 1/[σ^2^(*F*_o_^2^) + (0.0619*P*)^2^ + 0.131*P*] where *P* = (*F*_o_^2^ + 2*F*_c_^2^)/3
3463 reflections	(Δ/σ)_max_ < 0.001
175 parameters	Δρ_max_ = 0.12 e Å^−^^3^
0 restraints	Δρ_min_ = −0.18 e Å^−^^3^

### Special details

**Table:**

Geometry. All e.s.d.'s (except the e.s.d. in the dihedral angle between two l.s. planes) are estimated using the full covariance matrix. The cell e.s.d.'s are taken into account individually in the estimation of e.s.d.'s in distances, angles and torsion angles; correlations between e.s.d.'s in cell parameters are only used when they are defined by crystal symmetry. An approximate (isotropic) treatment of cell e.s.d.'s is used for estimating e.s.d.'s involving l.s. planes.
Refinement. Refinement of *F*^2^ against ALL reflections. The weighted *R*-factor *wR* and goodness of fit *S* are based on *F*^2^, conventional *R*-factors *R* are based on *F*, with *F* set to zero for negative *F*^2^. The threshold expression of *F*^2^ > σ(*F*^2^) is used only for calculating *R*-factors(gt) *etc*. and is not relevant to the choice of reflections for refinement. *R*-factors based on *F*^2^ are statistically about twice as large as those based on *F*, and *R*- factors based on ALL data will be even larger.

### Fractional atomic coordinates and isotropic or equivalent isotropic displacement parameters (Å^2^)

**Table:**

	*x*	*y*	*z*	*U*_iso_*/*U*_eq_	
O1	0.48715 (15)	0.63510 (5)	0.85500 (9)	0.0586 (3)	
N1	0.37002 (15)	0.53191 (5)	0.73822 (10)	0.0447 (3)	
N2	0.44565 (18)	0.70399 (5)	0.59706 (12)	0.0563 (3)	
N3	0.33493 (16)	0.64771 (5)	0.39424 (11)	0.0486 (3)	
C1	0.29597 (16)	0.49624 (6)	0.61429 (12)	0.0400 (3)	
C2	0.24489 (19)	0.42505 (6)	0.61944 (13)	0.0483 (3)	
H2A	0.2608	0.4045	0.7039	0.058*	
C3	0.1725 (2)	0.38552 (7)	0.50312 (15)	0.0549 (3)	
H3A	0.1400	0.3384	0.5089	0.066*	
C4	0.1472 (2)	0.41521 (7)	0.37579 (14)	0.0563 (4)	
H4A	0.0976	0.3879	0.2968	0.068*	
C5	0.19492 (19)	0.48452 (7)	0.36623 (13)	0.0491 (3)	
H5A	0.1776	0.5039	0.2806	0.059*	
C6	0.26991 (16)	0.52691 (6)	0.48450 (12)	0.0397 (3)	
C7	0.32629 (17)	0.59928 (6)	0.49100 (12)	0.0407 (3)	
C8	0.39572 (18)	0.63518 (6)	0.61455 (13)	0.0445 (3)	
C9	0.41477 (18)	0.59803 (6)	0.73647 (12)	0.0441 (3)	
C10	0.4073 (2)	0.70816 (7)	0.46497 (16)	0.0587 (4)	
H10A	0.4276	0.7491	0.4225	0.070*	
C11	0.2801 (2)	0.64045 (8)	0.24607 (13)	0.0545 (3)	
H11A	0.3224	0.5946	0.2258	0.065*	
H11B	0.3465	0.6763	0.2129	0.065*	
C12	0.0645 (2)	0.64739 (9)	0.16888 (15)	0.0639 (4)	
H12A	−0.0010	0.6097	0.2001	0.077*	
C13	−0.0102 (3)	0.71824 (10)	0.1988 (2)	0.0988 (7)	
H13A	−0.1452	0.7221	0.1466	0.148*	
H13B	0.0578	0.7559	0.1737	0.148*	
H13C	0.0098	0.7214	0.2948	0.148*	
C14	0.0257 (3)	0.63667 (16)	0.01661 (19)	0.1156 (9)	
H14A	−0.1083	0.6447	−0.0342	0.173*	
H14B	0.0591	0.5892	0.0012	0.173*	
H14C	0.1018	0.6694	−0.0132	0.173*	
C15	0.5158 (3)	0.59705 (9)	0.97911 (15)	0.0718 (5)	
H15A	0.5586	0.6290	1.0555	0.108*	
H15B	0.6111	0.5610	0.9900	0.108*	
H15C	0.3968	0.5756	0.9753	0.108*	

### Atomic displacement parameters (Å^2^)

**Table:**

	*U*^11^	*U*^22^	*U*^33^	*U*^12^	*U*^13^	*U*^23^
O1	0.0849 (7)	0.0443 (5)	0.0409 (5)	−0.0024 (4)	0.0151 (5)	−0.0067 (4)
N1	0.0532 (6)	0.0404 (5)	0.0403 (5)	−0.0004 (4)	0.0162 (4)	−0.0010 (4)
N2	0.0727 (8)	0.0350 (6)	0.0589 (7)	0.0006 (5)	0.0202 (6)	0.0013 (5)
N3	0.0575 (6)	0.0416 (6)	0.0460 (6)	0.0052 (5)	0.0174 (5)	0.0094 (4)
C1	0.0401 (6)	0.0382 (6)	0.0419 (6)	0.0013 (4)	0.0147 (5)	−0.0012 (5)
C2	0.0547 (7)	0.0422 (7)	0.0496 (7)	−0.0038 (5)	0.0205 (6)	0.0020 (5)
C3	0.0614 (8)	0.0403 (7)	0.0622 (9)	−0.0084 (6)	0.0209 (6)	−0.0056 (6)
C4	0.0642 (9)	0.0483 (7)	0.0514 (8)	−0.0045 (6)	0.0140 (6)	−0.0134 (6)
C5	0.0561 (7)	0.0476 (7)	0.0405 (6)	0.0040 (6)	0.0130 (5)	−0.0014 (5)
C6	0.0392 (6)	0.0380 (6)	0.0407 (6)	0.0046 (5)	0.0126 (5)	0.0005 (5)
C7	0.0434 (6)	0.0377 (6)	0.0405 (6)	0.0068 (5)	0.0140 (5)	0.0054 (5)
C8	0.0517 (7)	0.0339 (6)	0.0465 (7)	0.0051 (5)	0.0154 (5)	0.0002 (5)
C9	0.0504 (7)	0.0402 (6)	0.0402 (6)	0.0041 (5)	0.0139 (5)	−0.0038 (5)
C10	0.0725 (9)	0.0381 (7)	0.0644 (9)	0.0021 (6)	0.0229 (7)	0.0091 (6)
C11	0.0586 (8)	0.0614 (8)	0.0447 (7)	0.0030 (6)	0.0196 (6)	0.0128 (6)
C12	0.0580 (8)	0.0774 (10)	0.0539 (8)	0.0034 (7)	0.0167 (7)	0.0247 (7)
C13	0.0755 (12)	0.0790 (12)	0.1277 (18)	0.0253 (9)	0.0180 (11)	0.0352 (12)
C14	0.0779 (13)	0.207 (3)	0.0512 (10)	−0.0164 (14)	0.0093 (9)	0.0234 (13)
C15	0.1055 (13)	0.0641 (10)	0.0429 (8)	−0.0088 (8)	0.0223 (8)	−0.0056 (7)

### Geometric parameters (Å, °)

**Table:**

O1—C9	1.3553 (14)	C6—C7	1.4257 (16)
O1—C15	1.4281 (17)	C7—C8	1.3839 (16)
N1—C9	1.2955 (15)	C8—C9	1.4137 (17)
N1—C1	1.3870 (15)	C10—H10A	0.9300
N2—C10	1.3051 (19)	C11—C12	1.523 (2)
N2—C8	1.3825 (15)	C11—H11A	0.9700
N3—C10	1.3635 (17)	C11—H11B	0.9700
N3—C7	1.3796 (15)	C12—C14	1.522 (2)
N3—C11	1.4571 (17)	C12—C13	1.523 (3)
C1—C2	1.4046 (17)	C12—H12A	0.9800
C1—C6	1.4199 (16)	C13—H13A	0.9600
C2—C3	1.3626 (18)	C13—H13B	0.9600
C2—H2A	0.9300	C13—H13C	0.9600
C3—C4	1.3902 (19)	C14—H14A	0.9600
C3—H3A	0.9300	C14—H14B	0.9600
C4—C5	1.3705 (18)	C14—H14C	0.9600
C4—H4A	0.9300	C15—H15A	0.9600
C5—C6	1.4095 (16)	C15—H15B	0.9600
C5—H5A	0.9300	C15—H15C	0.9600
			
C9—O1—C15	116.66 (10)	N2—C10—N3	114.66 (12)
C9—N1—C1	118.43 (10)	N2—C10—H10A	122.7
C10—N2—C8	103.07 (11)	N3—C10—H10A	122.7
C10—N3—C7	105.82 (11)	N3—C11—C12	113.66 (11)
C10—N3—C11	124.15 (11)	N3—C11—H11A	108.8
C7—N3—C11	130.03 (11)	C12—C11—H11A	108.8
N1—C1—C2	117.04 (11)	N3—C11—H11B	108.8
N1—C1—C6	124.31 (11)	C12—C11—H11B	108.8
C2—C1—C6	118.65 (11)	H11A—C11—H11B	107.7
C3—C2—C1	121.28 (12)	C14—C12—C11	108.51 (14)
C3—C2—H2A	119.4	C14—C12—C13	112.37 (17)
C1—C2—H2A	119.4	C11—C12—C13	110.99 (14)
C2—C3—C4	120.33 (12)	C14—C12—H12A	108.3
C2—C3—H3A	119.8	C11—C12—H12A	108.3
C4—C3—H3A	119.8	C13—C12—H12A	108.3
C5—C4—C3	120.24 (12)	C12—C13—H13A	109.5
C5—C4—H4A	119.9	C12—C13—H13B	109.5
C3—C4—H4A	119.9	H13A—C13—H13B	109.5
C4—C5—C6	120.88 (12)	C12—C13—H13C	109.5
C4—C5—H5A	119.6	H13A—C13—H13C	109.5
C6—C5—H5A	119.6	H13B—C13—H13C	109.5
C5—C6—C1	118.63 (11)	C12—C14—H14A	109.5
C5—C6—C7	127.34 (11)	C12—C14—H14B	109.5
C1—C6—C7	114.03 (10)	H14A—C14—H14B	109.5
N3—C7—C8	104.80 (10)	C12—C14—H14C	109.5
N3—C7—C6	133.69 (11)	H14A—C14—H14C	109.5
C8—C7—C6	121.49 (11)	H14B—C14—H14C	109.5
N2—C8—C7	111.65 (11)	O1—C15—H15A	109.5
N2—C8—C9	129.72 (11)	O1—C15—H15B	109.5
C7—C8—C9	118.55 (11)	H15A—C15—H15B	109.5
N1—C9—O1	120.52 (11)	O1—C15—H15C	109.5
N1—C9—C8	123.15 (11)	H15A—C15—H15C	109.5
O1—C9—C8	116.31 (11)	H15B—C15—H15C	109.5
			
C9—N1—C1—C2	−178.62 (11)	C10—N2—C8—C7	−0.46 (15)
C9—N1—C1—C6	1.65 (18)	C10—N2—C8—C9	176.13 (14)
N1—C1—C2—C3	−179.42 (12)	N3—C7—C8—N2	0.34 (14)
C6—C1—C2—C3	0.32 (18)	C6—C7—C8—N2	178.58 (11)
C1—C2—C3—C4	−0.2 (2)	N3—C7—C8—C9	−176.68 (11)
C2—C3—C4—C5	0.1 (2)	C6—C7—C8—C9	1.57 (18)
C3—C4—C5—C6	−0.1 (2)	C1—N1—C9—O1	179.84 (11)
C4—C5—C6—C1	0.29 (19)	C1—N1—C9—C8	−1.44 (18)
C4—C5—C6—C7	179.85 (12)	C15—O1—C9—N1	1.81 (19)
N1—C1—C6—C5	179.35 (11)	C15—O1—C9—C8	−177.00 (13)
C2—C1—C6—C5	−0.37 (17)	N2—C8—C9—N1	−176.51 (13)
N1—C1—C6—C7	−0.27 (16)	C7—C8—C9—N1	−0.11 (19)
C2—C1—C6—C7	−179.99 (11)	N2—C8—C9—O1	2.3 (2)
C10—N3—C7—C8	−0.08 (14)	C7—C8—C9—O1	178.66 (11)
C11—N3—C7—C8	−179.63 (12)	C8—N2—C10—N3	0.41 (16)
C10—N3—C7—C6	−178.01 (13)	C7—N3—C10—N2	−0.22 (17)
C11—N3—C7—C6	2.4 (2)	C11—N3—C10—N2	179.36 (12)
C5—C6—C7—N3	−3.3 (2)	C10—N3—C11—C12	−99.77 (16)
C1—C6—C7—N3	176.32 (12)	C7—N3—C11—C12	79.71 (17)
C5—C6—C7—C8	179.09 (12)	N3—C11—C12—C14	−178.33 (15)
C1—C6—C7—C8	−1.33 (16)	N3—C11—C12—C13	57.73 (17)

### Hydrogen-bond geometry (Å, °)

**Table:**

*D*—H···*A*	*D*—H	H···*A*	*D*···*A*	*D*—H···*A*
C10—H10A···O1^i^	0.93	2.39	3.3052 (16)	169.

Symmetry codes: (i) *x*, −*y*+3/2, *z*−1/2.
